# Human Peritoneal Mesothelial Cell Death Induced by High-Glucose Hypertonic Solution Involves Ca^2+^ and Na^+^ Ions and Oxidative Stress with the Participation of PKC/NOX2 and PI3K/Akt Pathways

**DOI:** 10.3389/fphys.2017.00379

**Published:** 2017-06-13

**Authors:** Felipe Simon, Pablo Tapia, Ricardo Armisen, Cesar Echeverria, Sebastian Gatica, Alejandro Vallejos, Alejandro Pacheco, Maria E. Sanhueza, Miriam Alvo, Erico Segovia, Rubén Torres

**Affiliations:** ^1^Departamento de Ciencias Biologicas, Facultad de Ciencias Biologicas and Facultad de Medicina, Universidad Andres BelloSantiago, Chile; ^2^Millennium Institute on Immunology and ImmunotherapySantiago, Chile; ^3^Unidad de Paciente Critico, Hospital Clínico Metropolitano de La FloridaSantiago, Chile; ^4^Centro de Investigación y Tratamiento del Cancer, Facultad de Medicina, Universidad de ChileSantiago, Chile; ^5^Center for Excellence in Precision Medicine Pfizer, Pfizer ChileSantiago, Chile; ^6^Centro Integrativo de Biología y Química Aplicada, Universidad Bernardo OHigginsSantiago, Chile; ^7^Sección de Nefrología, Departamento de Medicina, Hospital Clínico Universidad de ChileSantiago, Chile; ^8^Facultad de Medicina, Instituto de Ciencias Biomédicas, Universidad de ChileSantiago, Chile

**Keywords:** peritoneal tissue, mesothelial cell, cell death, hypertonicity, dialysis

## Abstract

Chronic peritoneal dialysis (PD) therapy is equally efficient as hemodialysis while providing greater patient comfort and mobility. Therefore, PD is the treatment of choice for several types of renal patients. During PD, a high-glucose hyperosmotic (HGH) solution is administered into the peritoneal cavity to generate an osmotic gradient that promotes water and solutes transport from peritoneal blood to the dialysis solution. Unfortunately, PD has been associated with a loss of peritoneal viability and function through the generation of a severe inflammatory state that induces human peritoneal mesothelial cell (HPMC) death. Despite this deleterious effect, the precise molecular mechanism of HPMC death as induced by HGH solutions is far from being understood. Therefore, the aim of this study was to explore the pathways involved in HGH solution-induced HPMC death. HGH-induced HPMC death included influxes of intracellular Ca^2+^ and Na^+^. Furthermore, HGH-induced HPMC death was inhibited by antioxidant and reducing agents. In line with this, HPMC death was induced solely by increased oxidative stress. In addition to this, the cPKC/NOX2 and PI3K/Akt intracellular signaling pathways also participated in HGH-induced HPMC death. The participation of PI3K/Akt intracellular is in agreement with previously shown in rat PMC apoptosis. These findings contribute toward fully elucidating the underlying molecular mechanism mediating peritoneal mesothelial cell death induced by high-glucose solutions during peritoneal dialysis.

## Introduction

Peritoneal dialysis (PD) is a renal replacement therapy used to treat patients undergoing end-stage kidney disease. This extrarenal procedure for the depuration of toxins and solutes is based on the underlying physiology of peritoneal tissue, which is formed by a monolayer of mesothelial cells, submesothelial connective tissue, and mesothelial capillaries. Using diffusive and convective mechanisms, this tissue acts as a semipermeable membrane that allows the movement of water and solutes from peritoneal blood to the dialysis solution (Mortier et al., 2004; Goffin, 2008; González-Mateo et al., 2009; García-López et al., 2012). During PD treatment, a high-glucose hyperosmotic (HGH) solution is repeatedly administered into the peritoneal cavity to create an osmotic gradient favorable to the transport of water and solutes. Three types of HGH solutions are used to perform PD: 1.5% (345 mOsM), 2.5% (395 mOsM), and 4.25% (485 mOsM) dextrose. Of these, 4.25% dextrose is frequently used in cases of overload volume and in patients with chronic impaired ultrafiltration.

Several studies confirm that PD therapy is comparable to hemodialysis in terms of survival, blood depuration, and body fluid balance (Teixeira et al., 2015). However, PD is associated with functional and morphological changes in the peritoneal tissue that impair efficiency (Davies et al., 2001; Williams et al., 2002; Kim, 2009). Indeed, an elevated glucose concentration has devastating consequences on peritoneal tissue, inducing severe inflammation during early treatment and peritoneal mesothelial cell death and tissue fibrosis during later treatment (Krediet, 1999; Davies et al., 2001; Książek et al., 2007b; Kim, 2009; Yokoi et al., 2013).

Several studies support a PD-induced loss of mesothelial cells. For example, after 12 months of PD, 7% of patients exhibit a partial or total denudation of mesothelial cells from the mesothelial monolayer (Ishibashi et al., 2002; Tarng, 2002; Krediet and Struijk, 2013). Additionally, human peritoneal biopsies in patients that underwent PD for 14 months showed a total loss of mesothelial cells in 40% of cases and partial deficiency in 34% of cases (Van Biesen et al., 2006; Goffin, 2008; González-Mateo et al., 2009). The main feature of PD-induced peritoneal damage is decreased ultrafiltration capacity in association with neoangiogenesis and a submesothelial accumulation of extracellular matrix proteins (Davies et al., 2001; Williams et al., 2002; Mortier et al., 2004; Goffin, 2008). Furthermore, some research suggests that the HGH solutions used in PD may induce mesothelial cell death (Ishibashi et al., 2002; Tarng, 2002; Boulanger et al., 2004; Krediet and Struijk, 2013; Hung et al., 2014). However, the underlying molecular mechanism mediating peritoneal mesothelial cell death as induced by an HGH solution during PD is not well-studied.

Cell death is mediated by several factors, including intracellular calcium ([Ca^2+^]_i_) and sodium ([Na^+^]_i_) concentration changes, reactive oxygen species (ROS)-induced oxidative stress, kinase activities, and ion channel activations (Henriquez et al., 2008; González-Mateo et al., 2009; Becerra et al., 2011; Nuñez-Villena et al., 2011). Under aerobic conditions or in response to extracellular inducers, living organisms generate ROS through metabolic pathways. The generation of ROS frequently involves the production of the superoxide anion (${\text{O}}_{2}^{•-}$), hydrogen peroxide (H_2_O_2_), and the hydroxyl radical, resulting in increased intracellular oxidative stress. To maintain homeostasis, reducing agents such as catalase, glutathione S-transferase, and antioxidant molecules are produced to decrease oxidative stress (Dröge, 2002). It is well-accepted that patients subjected to PD using HGH solutions exhibit increased oxidative stress as a combined consequence of enhanced ROS generation and decreased antioxidant mechanisms (Taylor et al., 1992; Ha and Lee, 2000; Tarng, 2002).

It is currently accepted that the main source of ROS in non-phagocytic cells is the enzymatic complex nicotinamide adenine dinucleotide phosphate-oxidase (NAD(P)H oxidase) type 2 (NOX2; Li, 2003; Lee et al., 2004). The mechanism for inducing NAD(P)H oxidase activity is triggered by activation of the phospholipase C (PLC)/PKC and PI3K/Akt pathways. These pathways phosphorylate serine residues in several subunits of NAD(P)H oxidase (el Benna et al., 1994; Park and Babior, 1997; Dang et al., 1999; Lopes et al., 1999; Babior, 2002, 2004; Simon and Stutzin, 2008). Additionally, it has been reported in rat peritoneal mesothelial cell (RPMC) the participation of the PI3K/Akt pathway in RPMC death induced by high glucose (Kaifu et al., 2009), suggesting that a similar mechanism was implicated in human cells. However, it is unknown if these signaling pathways are involved in HGH solution-induced human peritoneal mesothelial cell (HPMC) death.

Therefore, the aim of this study was to determine the underlying mechanism mediating HGH solution-induced HPMC death. Our findings indicate that when exposed to a HGH solution, HMPC exhibits extensive cell death involving Ca^2+^ and Na^+^ ions and oxidative stress with the participation of the PLC/PKC/NOX2 and PI3K/Akt pathways. Taken together, these results provide novel information for more fully understanding the underlying molecular mechanism mediating peritoneal mesothelial cell death induced by a high-glucose solution during PD. The results of this report may be useful for improving current dialysis therapies.

## Materials and methods

### Primary human peritoneal mesothelial cell culture

Primary human peritoneal mesothelial cells (HPMC) were obtained from an overnight (8 h) peritoneal lavage of seven patients admitted to the Hospital Clínico Universidad de Chile. The patients were under 50 years of age, presented neither systemic inflammatory diseases nor peritonitis and hemoperitoneum episodes, and were non-diabetic. HPMC were obtained from patients that had initiated PD within the first month prior to sampling. The peritoneal lavage bags were stored at 4°C for 1 h, and then 200 mL were extracted from the bottom of each bag. Cells were pelleted by centrifugation at 3000 rpm for 5 min and then cultured with Earle's M199 medium with 10% FBS, 2% Biogro-2, 50 UI penicillin, and 50 μg/mL streptomycin in a 5%:95% CO_2_:air atmosphere at 37°C. Every 48 h, the culture medium was changed. After 10 days of culturing, a mesothelial cells monolayer was obtained for experimental use.

The cultures were rich in HPMC that contained no detectable levels of cell contaminants, such as leukocytes and fibroblasts. The initial culture confluences were 80–90%, and cultures were used up to five consecutive passages. Each culture was grown from individual effluents. Each experiment was performed using an individual culture, and the obtained results were included in respective graphs.

This study and respective protocol were approved by and carried out in accordance with recommendations from the Bioethics Commission of the Universidad de Chile, with written informed consent from all subjects. All subjects were required to provide written informed consent for study inclusion, in accordance with the Declaration of Helsinki.

### Experimental isotonic and high-glucose hypertonic solutions

HPMC monolayers were exposed to an isosmotic 300 mOsM solution (composed of 5.5 mM glucose and 1% BSA in a culture medium) and the following hyperosmotic solutions: 345 mOsM solution (composed of 45 mM glucose and 1% BSA in a culture medium), 395 mOsM solution (composed of 95 mM glucose and 1% BSA in a culture medium), and 485 mOsM solution (composed of 185 mM glucose and 1% BSA in a culture medium). Osmolarity was measured with a micro-osmometer (Advanced Instruments, Norwood, MA, USA).

### Cell death determinations

Lactate dehydrogenase release: Cell death was determined by measuring lactate dehydrogenase activity released into the culture medium. After cells were incubated with the solutions described above for 24 h, lactate dehydrogenase activity in cell supernatants was determined by a colorimetric end-point kit according to the manufacturer's instructions (Roche, USA). Lactate dehydrogenase activity was measured at 590 nm. A calibration curve was produced to ensure linearity in the range studied. Various independent experiments were performed in triplicate. The background was subtracted, and data were expressed as the fraction of maximum release measured in the presence of 1% Triton X-100 (Sigma, USA).

Propidium iodide (PI)/Annexin V-FITC double labeling: Cells were harvested by centrifugation at 800 × *g* for 10 min, and the pellet was suspended in 100 μL PBS. Next, cells were incubated with PI (10 μg/mL, Sigma) and Annexin V-FITC (BD Pharmingen, San Diego, CA) according to the manufacturer's protocol for 20 min at room temperature in the dark. The cells were then washed and analyzed immediately by flow cytometry (FACSCanto, BD Biosciences, San Jose, CA) The PI and Annexin V excitation/emission wavelengths used were 488/>610 nm and 488/515–545 nm, respectively. A minimum of 30,000 cells/sample were analyzed. PI intensity analysis was performed using the FACSDiva software version 4.1.1 (BD Biosciences).

PI staining: Cells were harvested by centrifugation at 800 × *g* for 5 min, and the pellet was suspended in 200 μL PBS. Afterwards, cells were stained with PI (10 μg/mL, Sigma) for 20 min at room temperature in the dark. Then, cells were washed, and DNA content was analyzed with a flow cytometry system (FACSCanto, BD Biosciences, San Jose, CA). The PI excitation/emission wavelength used was 488/>610 nm. A minimum of 10,000 cells/sample were analyzed. PI intensity analysis was performed using the FACSDiva software version 4.1.1 (BD Biosciences; Simon and Fernández, 2009; Simon et al., 2010).

All solutions used in the flow cytometry experiments (i.e., washing and testing) were acquired from BD Biosciences and used according to the manufacturer's instructions.

### Detection of intracellular Ca^2+^, Na^+^, potential membrane, and ROS

HPMC were treated with the HGH solution and then loaded with different fluorescent dyes. For Ca^2+^ determinations: Either 5 μM Fluo-3 or 15 μM Fura-Red were used. Both of these dyes are Ca^2+^ specific and increase (Fluo-3) or decrease (Fura-Red) in fluorescent emission intensity upon binding Ca^2+^ (Nuñez-Villena et al., 2011). The Fluo-3 and Fura-Red excitation/emission wavelengths used were 488/533 and 488/>640 nm, respectively. For Na^+^ determinations: Dying procedures used 5 μM CoroNa Green-AM, a specific Na^+^ dye that increases in fluorescent emission intensity upon binding Na^+^ (Becerra et al., 2011). The CoroNa Green-AM excitation/emission wavelength used was 488/515–560 nm. For membrane-potential measurements cells: The cell-membrane depolarization indicator bis-(1,3-dibutylbarbituric acid) trimethine oxonol [DiBAC_4_(3); (Becerra et al., 2011)] was used. A region of interest used to measure membrane potential was identified in the live cell subpopulation, in accordance with previous descriptions (Becerra et al., 2011). The DiBAC_4_(3) excitation/emission wavelength used was 488/510 nm. For ROS measurements: Either 5 μM DCF (2′,7′-dichlorodihydrofluorescein diacetate [H_2_DCFDA]) or 10 μM DHE (dihydroethidium) were used. These two florescent indicators exhibit increased fluorescent emission intensity upon binding ROS. The DCF and DHE excitation/emission wavelengths used were 488/520 and 488/>580 nm, respectively. All dyes were obtained from Invitrogen.

Dyes were added for 15–30 min at room temperature in the dark and then washed three times before measuring [except DiBAC_4_(3); (Becerra et al., 2011)]. The labeled cells were then analyzed immediately by flow cytometry (FACSCanto, BD Biosciences, San José, CA). A minimum of 10,000 cells/sample were analyzed. Live cells were counted. Cellular dye intensity analysis was performed using the FACSDiva software v4.1.1 (BD Biosciences). All solutions used in the flow cytometry experiments (i.e., washing and testing) were acquired from BD Biosciences and used according to the manufacturer's instructions.

### Small interfering RNA transfection

SiGENOME siRNA against NOX2 (siNOX2) and non-targeting siRNA (siCTRL) were used as control sequences (Dharmacon, Lafayette, CO). Transfections were performed using the DharmaFECT 4 transfection reagent (Dharmacon) according to manufacturer protocols.

### Western blotting for NOX2

Cells transfected with siNOX2 or siCTRL were lysed, and proteins were extracted. Whole cell extracts were subjected to 10% SDS-PAGE. Resolved proteins were transferred to a nitrocellulose membrane, blocked, and then incubated overnight with the anti-NOX2 antibody (Abcam). Tubulin was used as a loading control (Sigma). Protein content was determined by densitometric scanning of immunoreactive bands, and intensity values were obtained through the densitometry of individual bands as compared with tubulin and normalized against siCTRL.

### Reagents and solutions

The following were purchased from Calbiochem (USA): non-selective calcium channel blocker 2-aminoethoxydiphenyl borate (2-APB, 10 μM), L-type Ca^2+^ channel blocker nifedipine (1 μM), non-selective sodium channel blocker (Ambroxol, 1 μM), Na^+^ channel blocker amiloride (1 μM), cell-permeable calcium chelator BAPTA-AM (5 μM, pulse for 4 h), Catalase (10 μM), dipheniliodonium (10 μM), apocynin (1 mM), Rotenone (1 μM), Allopurinol (10 μM), 2-[1-(3-dimethylaminopropyl)-1H-indol-3-yl]-3-(1H-indol-3-yl)maleimide (BIM, 50 μM), Gö 6976 (1 μM), N-acetylcysteine (NAC, 5 mM), U73122 (10 μM), and gluthathione (GSH and GSSG, 10 mM). The following were purchased from Tocris (USA): L-NG-arginine methyl ester hydrochloride (L-NAME, 10 μM), the NO scavenger 2-Phenyl-4,4,5,5-tetramethylimidazoline-1-oxyl-3-oxide (PTIO, 10 μM), 2-(4-Morpholinyl)-8-phenyl-4H-1-benzopyran-4-one hydrochloride (Ly294002, 20 μM), 1,5-Dihydro-5-methyl-1-β-D-ribofuranosyl-1,4,5,6,8-pentaazaacenaphthylen-3-amine (API-2 10 μM), G–protein inhibitor (SCH 202676 hydrobromide, 10 μM), and tyrosine-kinase receptor inhibitor (AG 18, 50 μM). All reagents were added 1 h before HGH solution exposure and were maintained during the experiments. All inhibitors and blockers were tested to ensure efficiency before use.

Experiments were performed in Earle's M199 medium solution with 1% FBS, 2% Biogro-2, 50 UI penicillin, and 50 μg/mL streptomycin containing the following (in mM): NaCl 130, KCl 2.7, Na_2_HPO_4_ 10, KH_2_PO_4_ 1.8, CaCl_2_ 2.5, MgCl_2_ 1, HEPES 5. Experiments were performed at a pH 7.4, as adjusted with HCl/NaOH in a 5%:95% CO_2_:air atmosphere at 37°C. For the Ca^2+^-free solution, CaCl_2_ was not added and was replaced with MgCl_2_. For the Na^+^-free solution, NaCl was replaced with NMDG-Cl.

### Statistical analysis

All results are presented as the means ± *SD*. Statistical differences were assessed by a student's *t*-test (Mann–Whitney), one-way analysis of variance (ANOVA; or the non-parametric Kruskal–Wallis method) followed by Dunn's *post-hoc* test, or two-way ANOVA followed by Tukey's *post-hoc* test, as respectively indicated in the figure legends. Differences were considered significant at *p* < 0.05.

## Results

### High-glucose hypertonic solution induces human peritoneal mesothelial cell death

**Figure 1:**
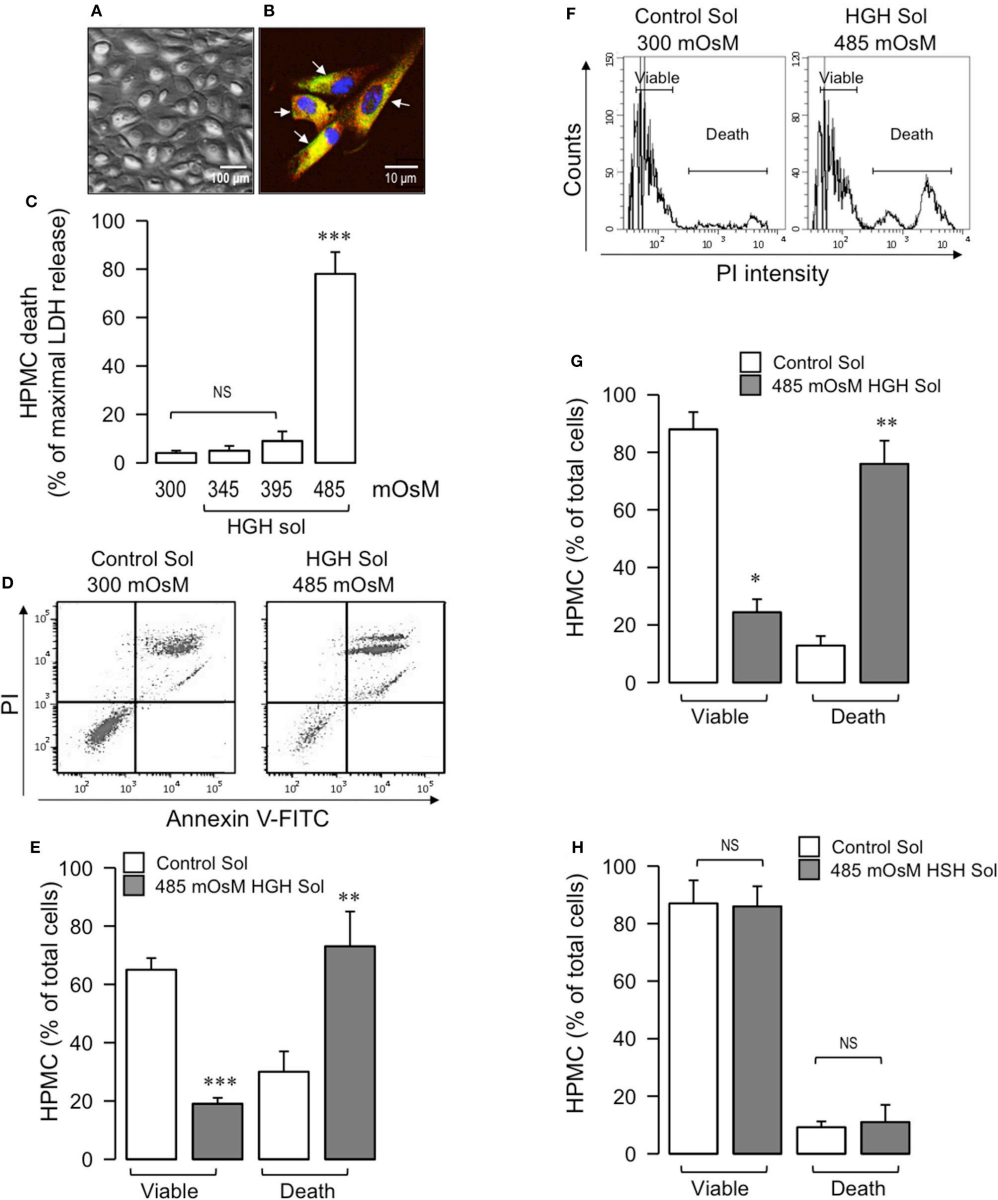
HGH solution induces HPMC death. Primary HPMC were obtained from patients in their first month of PD. **(A)** Representative phase-contrast microscopy image of second-pass HPMC monolayer depicting cobblestone morphology. **(B)** Representative second-pass HPMC image subjected to immunofluorescence, detecting pancitokeratin (red), and GLUT-1 (green, arrow). Nuclei were stained using DAPI. **(C)** HPMC were exposed to 300, 345, 395, or 485 mOsM HGH solutions for 24 h, and cell death was determined by means of LDH release. Statistical differences were assessed by one-way ANOVA (or the non-parametric Kruskal–Wallis method) followed by Dunn's *post-hoc* test. ^***^P < 0.001 against control (Ctrl) condition. NS: non-significant. Graph bars show the mean ± *SD* (*N* = 5 independent cultures). **(D)** Representative PI and Annexin V-FITC double staining dot-plot of HPMC in control (300 mOsM) condition or exposed to 485 mOsM HGH solution for 24 h. Viable (no staining, lower-left quadrant) and dead (Annexin V-FITC staining only, lower-right quadrant and PI and Annexin V-FITC double staining, upper-right quadrant) cells are shown. **(E)** Viable and dead HPMC in control (open bars) condition or exposed to 485 mOsM HGH solution (gray bars) recorded from experiments as depicted in **(D)**. Statistical differences were assessed by two-way ANOVA followed by Tukey's *post-hoc* test. ^**^P < 0.01 and ^***^P < 0.001 against control condition. Graph bars show the mean ± *SD* (*N* = 5–6 independent cultures). **(F)** Representative DNA content histograms of HPMC in control (300 mOsM) condition or exposed to 485 mOsM HGH solution for 24 h. Viable and dead HPMC were determined as PI binds to DNA, and PI fluorescence is proportional to DNA content. Viable cells present a low basal PI fluorescence, whereas dead cells bind higher levels of PI. **(G)** Viable and dead HPMC in control (open bars) condition or exposed to 485 mOsM HGH solution (gray bars) recorded from experiments as depicted in **(F)**. Statistical differences were assessed by two-way ANOVA followed by Tukey's *post-hoc* test. ^*^P < 0.05 and ^**^P < 0.01 against control condition. Graph bars show the mean ± *SD* (*N* = 6 independent cultures). **(H)** Viable and dead HPMC in control (open bars) condition or exposed to 485 mOsM HSH solution (gray bars) recorded from experiments similar to those shown in **(F)**. NS, non-significant. Graph bars show the mean ± *SD* (*N* = 6 independent cultures).

The HPMC had a round, short-spindle morphology with a cobblestone appearance and formed a confluent monolayer (Figure 1A). In accordance with previous descriptions for wild-type mesothelial cells, the HPMC expressed the proteins pancitokeratin and GLUT-1 (Figure 1B). Furthermore, HPMC monolayer viability was preliminarily evaluated through incorporation of the vital dye calcein (not shown).

Following this, the total HPMC death induced by HGH exposure was determined. The HPMC monolayer was exposed to either an isosmotic solution (300 mOsM), as a control, or to HGH solutions of 345, 395, and 485 mOsM for 24 h, after which lactate dehydrogenase release was measured. HPMC incubated with the 485 mOsm HGH solution exhibited significant cell death, measured as lactate dehydrogenase release, whereas incubation with the 345 and 395 mOsM HGH solutions showed no differences compared to the control isosmotic solution (Figure 1C). Additionally, experiments were performed that challenged HPMC to either isosmotic or HGH solutions while simultaneously evaluating PI and annexin V-FITC staining. Cells exposed to HGH incorporated PI and bound annexin V-FITC, indicating that the HGH solution induced extensive HPMC death (Figures 1D,E). Similar results were obtained when measuring HGH-induced cell death via the DNA content detected by PI incorporation. HPMC exposed to HGH showed strongly increased PI incorporation in the death population, along with decreased PI incorporation in the viable HPMC region (Figures 1F,G). These results suggest that at 485 mOsm, the hyperosmotic solution efficiently generates extensive HPMC cell death.

To test the relevance of glucose in the hypertonic solution, experiments using sorbitol as the hypertonic compound were performed in the absence of any glucose. The results demonstrated that the high-sorbitol hypertonic solution did not induce HPMC death, suggesting that HGH-induced HPMC death is dependent on glucose (Figure 1H).

### High-glucose hypertonic solution-induced human peritoneal mesothelial death is dependent on Ca^2+^ and Na^+^ ion channels

**Figure 2:**
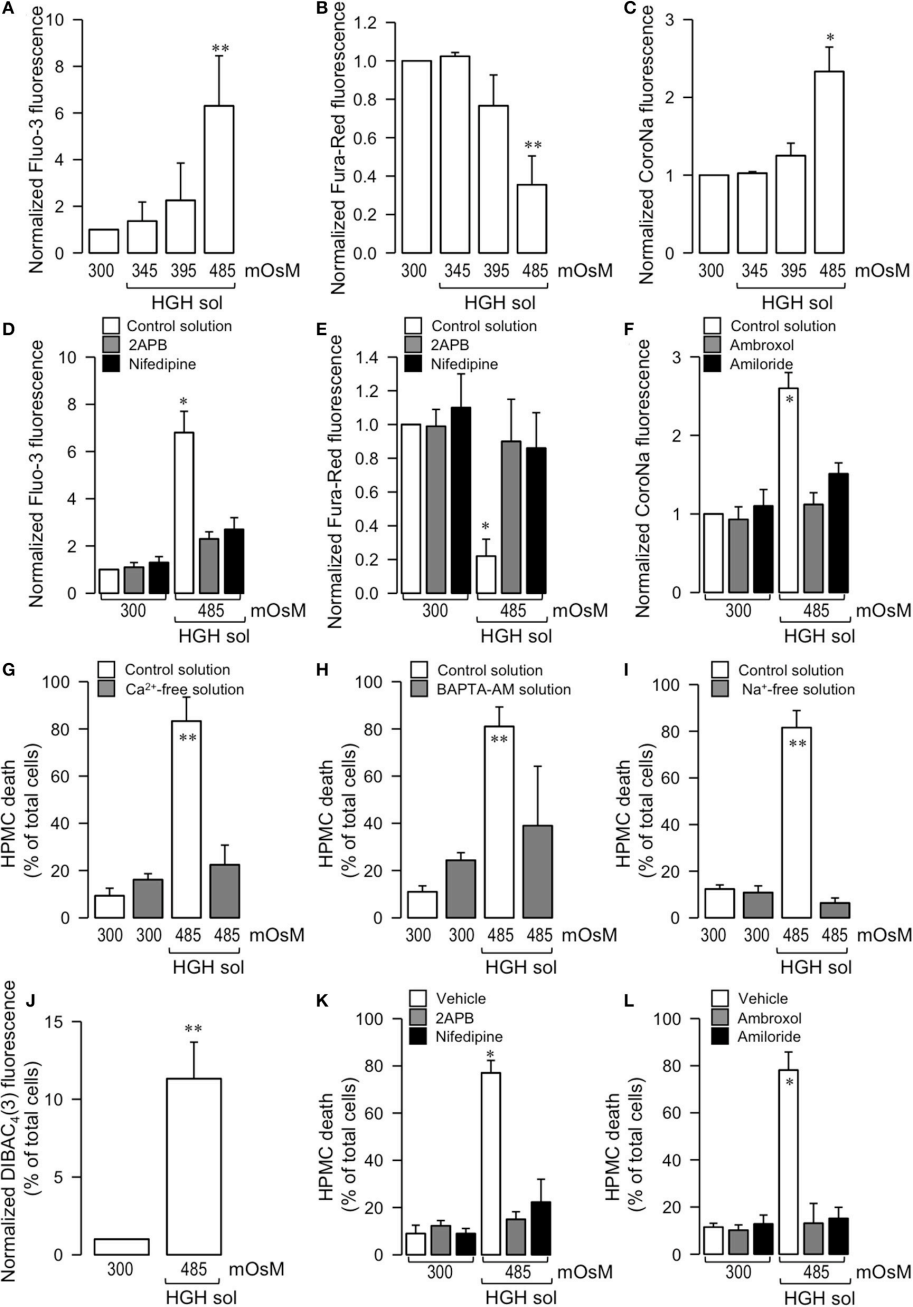
Potential participation of Ca^2+^ and Na^+^ ions in HGH solution-induced HPMC death. HPMC were exposed to a control (300 mOsM) condition or exposed to 345, 395, or 485 mOsM HGH solutions for 24 h. Then, both intracellular Ca^2+^ **(A,B)** and Na^+^ **(C)** levels were determined by means of the Ca^2+^ sensitive dye Fluo-3 (5 μM) **(A)** and Fura-Red (15 μM) **(B)** and the Na^+^-sensitive dye CoroNa Green-AM (5 μM) **(C)**. Statistical differences were assessed by one-way ANOVA (or the non-parametric Kruskal–Wallis method) followed by Dunn's *post-hoc* test. ^*^P < 0.05 and ^**^P < 0.01 against control condition (300 mOsM). Graph bars show the mean ± *SD* (*N* = 5 independent cultures). HPMC were cultured in the absence (control solution, open bars) or presence of 10 μM 2-APB (gray bars, **D,E**), 1 μM nidefipine (filled bars, **D,E**), 1 μM ambroxol (gray bars, **F**), and 1 μM amiloride (filled bars, **F**), and then exposed to the control (300 mOsM) condition or to the 485 mOsM HGH solution for 24 h. Then, both intracellular Ca^2+^ **(D,E)** and Na^+^ **(F)** levels were determined by means of the Ca^2+^ sensitive dye 5 μM Fluo-3 **(D)** and 15 μM Fura-Red **(E)** and the Na^+^-sensitive dye 5 μM CoroNa Green-AM **(F)**. Statistical differences were assessed by two-way ANOVA followed by Tukey's *post-hoc* test. ^*^P < 0.05 against control condition (300 mOsM). Graph bars show the mean ± *SD* (*N* = 7 independent cultures). HPMC death was determined in cells cultured in the absence (unfilled bars) or presence (filled bars) of a Ca^2+^-free solution (4 h pulse) **(G)**, BAPTA-AM solution (5 μM, 4 h pulse) **(H)**, and a Na^+^-free solution **(I)**, and exposed to the control (300 mOsM) condition or the 485 mOsM HGH solution for 24 h. Statistical differences were assessed by two-way ANOVA followed by Tukey's *post-hoc* test. ^**^P < 0.01 against control condition (300 mOsM). Graph bars show the mean ± *SD* (*N* = 7 independent cultures). Cell membrane depolarization was measured by the indicator DiBAC_4_(3) in cells exposed to the control (300 mOsM) condition or the 485 mOsM HGH solution **(J)**. Statistical differences were assessed by the student's *t*-test (Mann-Whitney). ^**^P < 0.01 against control condition (300 mOsM). Graph bars show the mean ± *SD* (*N* = 8 independent cultures). HPMC death was determined in cells cultured in the absence (control solution, unfilled bars) or presence of 10 μM 2-APB (gray bars, **K**), 1 μM nidefipine (filled bars, **K**), 1 μM ambroxol (gray bars, **L**), and 1 μM amiloride (filled bars, **L**), and then exposed to the control (300 mOsM) condition or the 485 mOsM HGH solution for 24 h. Statistical differences were assessed by two-way ANOVA followed by Tukey's *post-hoc* test. ^*^P < 0.05 against control condition (300 mOsM). Graph bars show the mean ± *SD* (*N* = 6 independent cultures). All reagents were added 1 h before HGH solution exposure and were maintained during the experiments.

Considering that intracellular ion levels can modulate cell death in almost all cell types, focus was placed on determining if HGH-induced mesothelial cell death was mediated by external ions. Calcium levels were independently determined with two different calcium-sensitive fluorescent dyes, one that increases (Fluo-3) and another that decreases (Fura-Red) in fluorescence upon calcium binding. Additionally, intracellular sodium changes were measured using the Na^+^-sensitive fluorescence probe CoroNa. HPMC exposed to the 485 mOsm HGH solution exhibited a significant Fluo-3 fluorescence increase and Fura-Red decrease, whereas at 345 and 395 mOsM HGH, no differences were observed as compared to the control isosmotic solution, indicating the occurrence of an intracellular Ca^2+^ increase (Figures 2A,B). Similarly, HGH-treated HPMC showed an increase in CoroNa fluorescence, indicating an increase in intracellular sodium (Figure 2C).

To test if intracellular Ca^2+^ and Na^+^ influxes were facilitated by an ion channel-mediated influx, experiments were performed using calcium and sodium channels blockers.

A non-selective Ca^2+^ channel blocker, 2-APB, effectively inhibited the fluorescence change of Fluo-3 and Fura-Red (Figures 2D,E, gray bars). Similar results were obtained using the L-type Ca^2+^ channel blocker nifedipine (Figures 2D,E, filled bars). Likewise, the sodium channel blockers, ambroxol and amiloride, abolished the CoroNa fluorescence increase (Figure 2F, gray and filled bars, respectively).

Interestingly, the reduction of either external Ca^2+^ or Na^+^ effectively prevented HGH-induced HPMC death. An initial 4 h pulse of extracellular Ca^2+^ absence effectively decreased HPMC death as induced by the HGH solution (Figure 2G). Similarly, a 4 h pulse of intracellular Ca^2+^ chelating was also able to reduce HGH-induced HPMC death (Figure 2H). Furthermore, a culture medium depleted of Na^+^ was prepared, thus keeping osmolarity and tonicity constant, with Na^+^ replaced with the non-permeant cation NMDG^+^. HPMC cultured in a Na^+^-free medium were resistant to HGH-induced HPMC death (Figure 2I). Considering that Na^+^ influx-dependent cell death is often induced by plasma membrane depolarization, assessments were also performed to determine if the HGH solution modified HPMC membrane potential. The HGH solution increased the fluorescence of the membrane potential fluorescent indicator DIBAC_4_(3), suggesting that the HGH solution induced HPMC membrane depolarization (Figure 2J).

Next, tests were performed to evaluate if HGH solution-induced HPMC death was dependent on ion channel activity. For this, HPMC were exposed to either Ca^2+^ or Na^+^ channel blockers. Preincubation with 2-APB, a non-specific calcium channel blocker, prevented HPMC death as induced by HGH exposure (Figure 2K, open bars). Furthermore, HPMC incubated in the presence of nifedipine, an L-type Ca^2+^ channel blocker, and exposed to HGH solution did not exhibit significant cell death (Figure 2K, full bars). Besides this, cells incubated with a non-specific sodium channel blocker, ambroxol, effectively resisted HGH solution-induced HPMC death (Figure 2L, open bars). In addition to this, cells pre-incubated with a more specific Na^+^ channel blocker, amiloride, were also resistant to the HGH solution challenge (Figure 2L, full bars).

### High-glucose hypertonic solution-induced human peritoneal mesothelial cell death is dependent on the generation of intracellular oxidative stress

**Figure 3:**
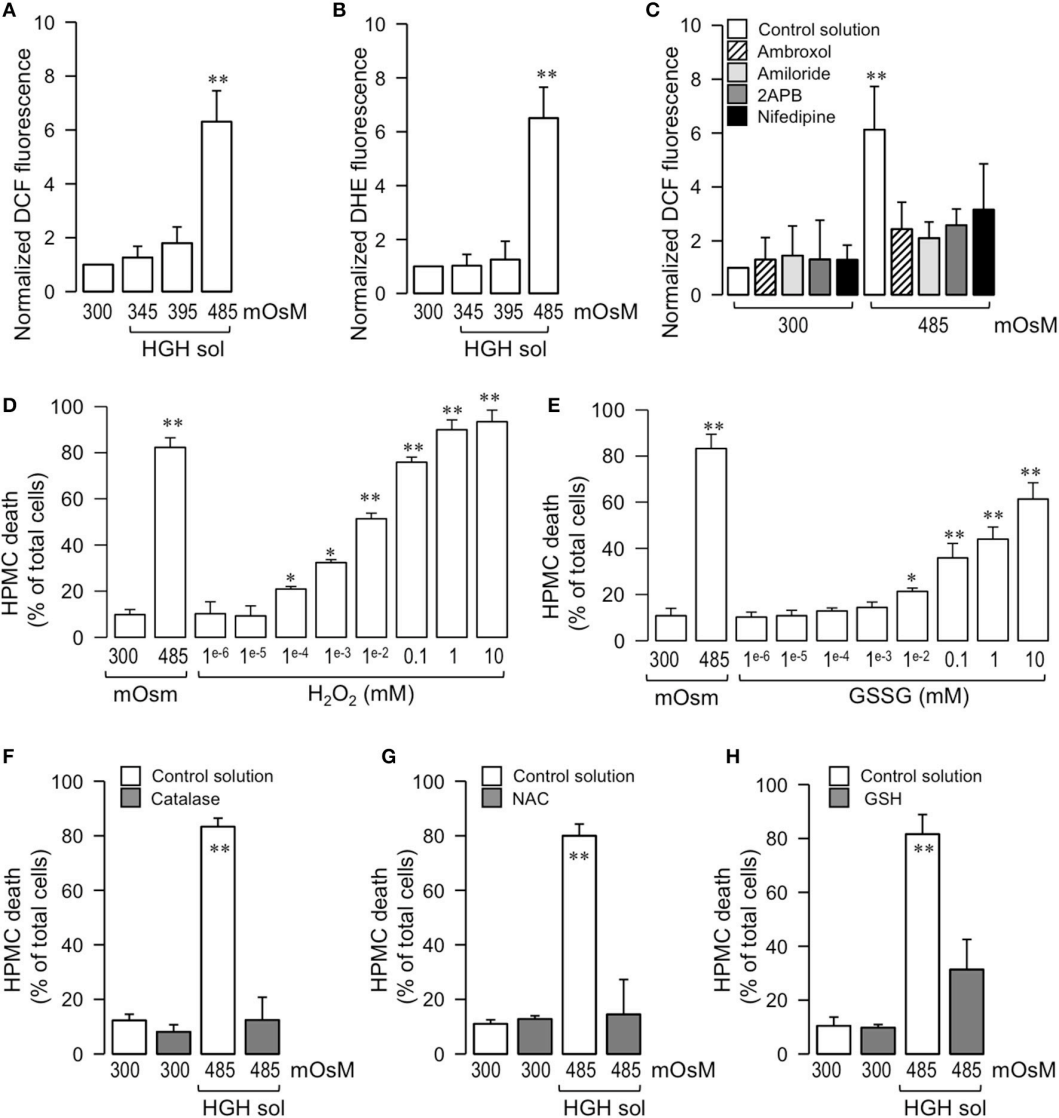
HGH solution-induced HPMC death is dependent on the generation of intracellular oxidative stress. HPMC intracellular ROS was determined by means of the ROS-sensitive dye DCF (5 μM) **(A)** and DHE (10 μM) **(B)** in HPMC exposed to the control (300 mOsM) condition or to 345, 395, or 485 mOsM HGH solutions for 24 h. Statistical differences were assessed by one-way ANOVA (or the non-parametric Kruskal–Wallis method) followed by Dunn's *post-hoc* test. ^**^P < 0.01 against control condition (300 mOsM). Graph bars show the mean ± *SD* (*N* = 9 independent cultures). **(C)** HPMC intracellular ROS was determined by the ROS-sensitive dye DCF (5 μM) in the absence (control solution, unfilled bars) or presence of 10 μM 2-APB, 1 μM nidefipine, 1 μM ambroxol, and 1 μM amiloride, and then exposed to the control (300 mOsM) condition or to the 485 mOsM HGH solution for 24 h. Statistical differences were assessed by two-way ANOVA followed by Tukey's *post-hoc* test. ^*^P < 0.05 against control condition (300 mOsM). Graph bars show the mean ± *SD* (*N* = 6 independent cultures). All reagents were added 1 h before HGH solution exposure and were maintained during the experiments. HPMC death was determined in cells cultured in the presence 10 mM–1 nM H_2_O_2_ **(D)** and 10 mM–1 nM GSSG **(E)**, and then exposed to the control (300 mOsM) condition for 24 h. Statistical differences were assessed by one-way ANOVA (or the non-parametric Kruskal–Wallis method) followed by Dunn's *post-hoc* test. ^*^P < 0.05 and ^**^P < 0.01 against control condition (300 mOsM). Graph bars show the mean ± *SD* (*N* = 4 independent cultures). HPMC death was determined in cells cultured in the absence (unfilled bars) or presence (gray bars) of either Catalase (10 μM) **(F)**, N-acetylcysteine (NAC, 5mM) **(G)** or GSH (10 mM) **(H)**, and then exposed to the control (300 mOsM) condition or the 485 mOsM HGH solution for 24 h. Statistical differences were assessed by two-way ANOVA followed by Tukey's *post-hoc* test. ^*^P < 0.05 and ^**^P < 0.01 against control condition (300 mOsM). Graph bars show the mean ± *SD* (*N* = 4 independent cultures).

Taking into consideration that ROS family members are involved in several processes of cell death, the involvement of these reactive molecules in HGH-induced HPMC death was analyzed. HPMC exposed to 485 mOsM HGH showed a strong increase in oxidative stress, which was measured through the fluorescence of two ROS-sensitive fluorescent probes, DCF, which is selective to H_2_O_2_, and DHE, which is more selective to ${\text{O}}_{2}^{•-}$. In turn, HPMC treated with the 300 mOsM solution and with the 345 and 395 mOsM HGH solutions did not exhibit any changes in DCF or DHE fluorescence (Figures 3A,B, respectively). Interestingly, HGH solution-induced ROS generation was significantly decreased when cells were treated with Ca^2+^ or Na^+^ channel blockers (Figure 3).

To test the participation of ROS in HPMC death induced by HGH exposure, dose-response studies were performed. HPMC exposed to H_2_O_2_ alone resulted in cell death comparable to that detected using the HGH solution (Figure 3C). Interestingly, the oxidized form of the endogenous molecule glutathione (GSSG) generated HPMC death that was similar to that produced using the HGH solution (Figure 3D). Following these results, tests were carried out to assess if inhibiting the ROS burst produced by the HGH solution challenge would decrease HGH-induced HPMC death. For this, HGH-treated HPMC were pre-incubated with a catalase enzyme to reduce H_2_O_2_ contents, and then cell death was measured (Dröge, 2002; Varela et al., 2004). The catalase pre-treatment significantly decreased HGH solution-induced HPMC death, suggesting the participation of H_2_O_2_ in this process (Figure 3E). Similar results were observed using both the antioxidant agent NAC (Figure 3F) and the reduced form of the endogenous reducing molecule glutathione (GSH; Figure 3G).

### High-glucose hypertonic solution-induced human peritoneal mesothelial cell death is mediated through NOX2 activity and nitric oxide

**Figure 4:**
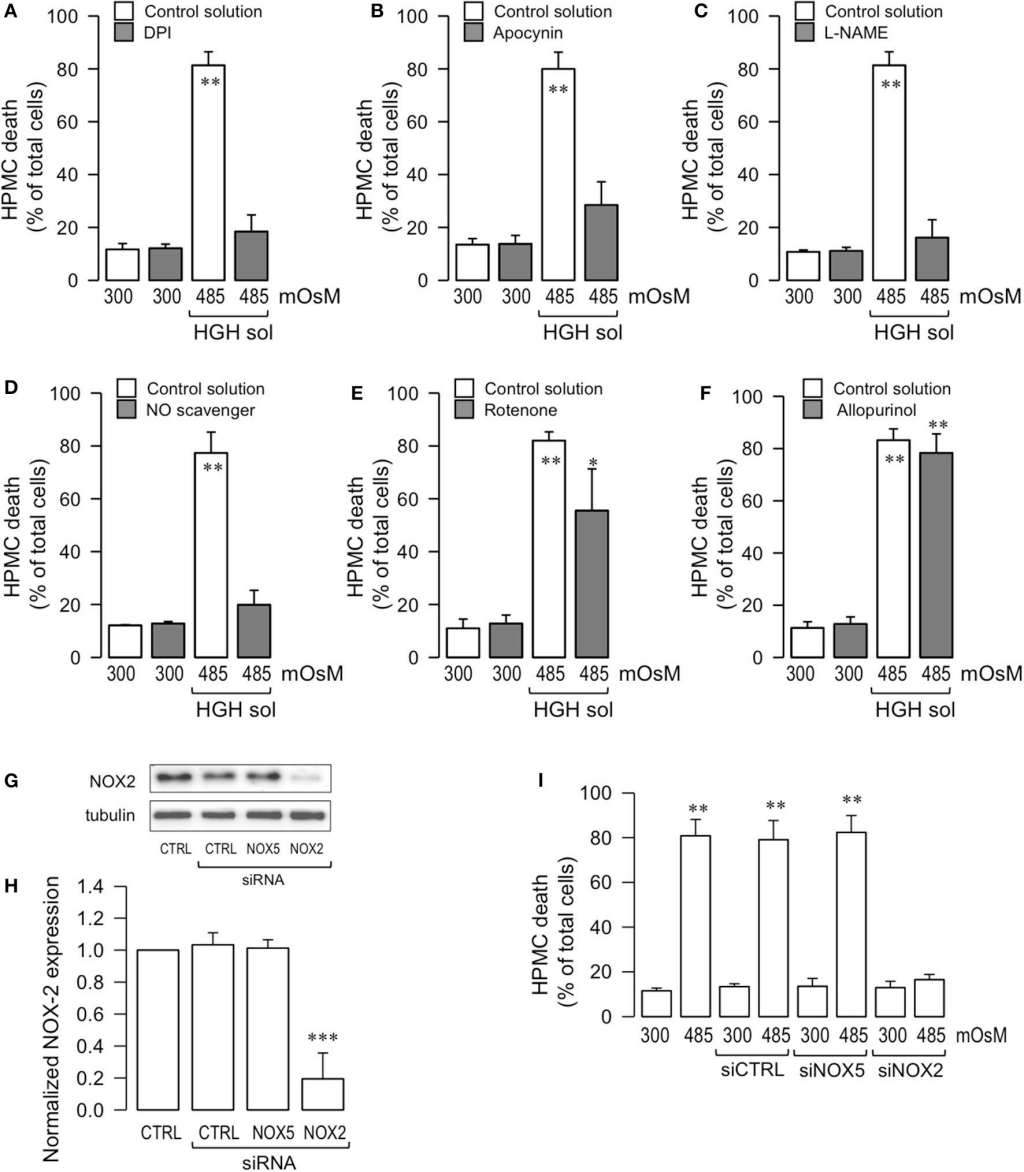
HGH solution-induced HPMC death is mediated through NOX2 activity and nitric oxide. HPMC death was determined in cells cultured in the absence (open bars) or presence (gray bars) of either DPI (10 μM) **(A)**, apocynin (1 mM), **(B)**, L-NAME (1 μM) **(C)**, NO scavenger (1 μM) **(D)**, rotenone (1 μM) **(E)**, and allopurinol (1 μM) **(F)**, and then exposed to the control (300 mOsM) condition or the 485 mOsM HGH solution for 24 h. Statistical differences were assessed by two-way ANOVA followed by Tukey's *post-hoc* test. ^*^P < 0.05 and ^**^P < 0.01 against control condition (300 mOsM). Graph bars show the mean ± *SD* (*N* = 4 independent cultures). **(G)** Representative image from western blot experiments performed for NOX2 expression downregulation by siRNA. Endothelial cells were transfected with a specific siRNA against the NOX2 isoform (siRNA-NOX2) or a non-targeting siRNA (siRNA-CTRL). A non-related siRNA against NOX5 (siRNA-NOX5) was used as a control. **(H)** Densitometric analyses from several experiments, as shown in **(G)**. Protein levels were normalized against tubulin, and the data are expressed relative to the control (CTRL) condition. Statistical differences were assessed by one-way ANOVA (or the non-parametric Kruskal–Wallis method) followed by Dunn's *post-hoc* test. ^***^P < 0.001 against control condition (non-transfected). Graph bars show the mean ± *SD* (*N* = 4 independent cultures). **(I)** HPMC death was determined in cells transfected with siRNA-CTRL, siRNA-NOX2, and siRNA-NOX5, and then exposed to the control (300 mOsM) condition or the 485 mOsM HGH solution for 24 h. Statistical differences were assessed by one-way ANOVA (or the non-parametric Kruskal–Wallis method) followed by Dunn's *post-hoc* test. ^**^P < 0.01 against control condition (300 mOsM, non-transfected). Graph bars show the mean ± *SD* (*N* = 5 independent cultures).

Since it is well-accepted that cell death is often induced by an imbalance in the sources of intracellular oxidative stress, experiments were performed to investigate if the ROS generating enzyme NOX was involved in HGH-induced HPMC death. HPMC were pre-incubated with the non-selective NOX inhibitor dipheniliodonium (DPI), and cells were challenged with HGH. The DPI treatment abolished HGH-induced HPMC death (Figure 4A). Likewise, the NOX inhibitor, apocynin, also decreased HPMC death as induced by the HGH solution (Figure 4B), suggesting the participation of NOX in HGH-induced HPMC death.

It has been reported that NO production is stimulated by exposure to high glucose concentrations (Liao et al., 2010; Hua et al., 2012; Zhai et al., 2013). Additionally, NO is involved in necrosis and apoptosis for a number of cell types, likely due to the contributions of NO to ROS formation (Azuara et al., 2003; Borutaite and Brown, 2003; Marriott et al., 2004). Therefore, assessments were carried out to determine if NO generation participated in the HPMC death induced by HGH conditions. HPMC were pre-incubated with L-NAME, an inhibitor of NOS, and cells were then exposed to the HGH solution to determine cell death. L-NAME-treated HPMC were resistant to HGH-induced HPMC death (Figure 4C). Similarly, the use of a NO scavenger (PTIO) also evidenced protective effects to the HGH challenge (Figure 4D), indicating that HGH-induced HPMC death is dependent on the NO generated from NOS activity.

Considering that other intracellular sources of ROS, such as mitochondria and xanthine oxidase, are involved in cell death (Dröge, 2002), experiments were performed using a mitochondrial uncoupler, rotenone, and a xanthine oxidase inhibitor, allopurinol. Rotenone treatment was modestly effective in inhibiting cell death induced by the HGH solution challenge (Figure 4E). Furthermore, incubation with allopurinol had no effect on inhibiting HPMC death as induced by the HGH solution (Figure 4F).

Several isoforms of NOX have been reported, such as NOX1 to NOX5. The NOX2 isoform is primarily linked to pathological processes. Although the results obtained using DPI and apocynin suggested the potential contribution of NOX2 in HGH-induced HPMC death, this result was far from being conclusive. Therefore, to more fully test the participation of NOX2, HPMC was transfected with a specific siRNA against NOX2 expression (siNOX2). NOX2 expression was severely decreased by siNOX2 transfection, demonstrating the efficiency of this siRNA (Figures 4G,H). Importantly, non-targeting siRNA (siCTRL) and a non-related siRNA (siRNA against NOX5) induced no changes in NOX2 expression (Figures 4G,H). Of note, HGH-induced HPMC death decreased when siNOX2 was transfected, indicating that this NOX isoform is crucial for ROS-mediated HGH-induced HPMC death (Figure 4I).

### High-glucose hypertonic solution-induced human peritoneal mesothelial cell death is mediated through the G-Protein/PLC/cPKC and tyrosine-kinase receptor/PI3K/Akt pathways

**Figure 5:**
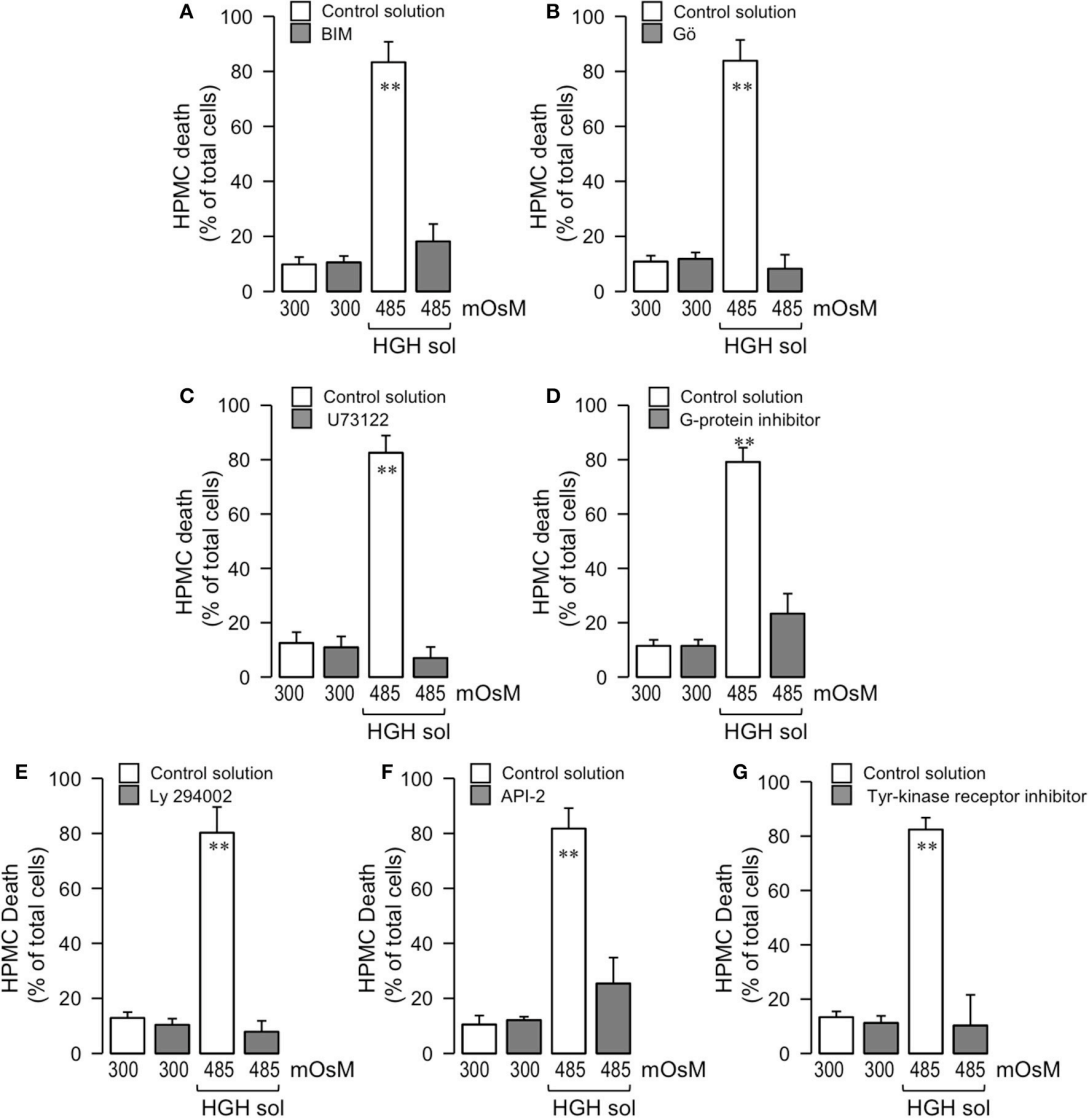
High-glucose hypertonic solution-induced human peritoneal mesothelial cell death is mediated through the G-protein/PLC/cPKC and tyrosine-kinase receptor/PI3K/Akt pathways. HPMC death was determined in cells cultured in the absence (unfilled bars) or presence (gray bars) of either BIM (50 μM) **(A)**, Gö (1 μM) **(B)**, U73122 (10 μM) **(C)**, G-protein inhibitor SCH 202676 (10 μM) **(D)**, Ly 294002 (20 μM) **(E)**, Akt inhibitor API-2 (10 μM) **(F)**, or tyrosine kinase receptor inhibitor AG18 (50 μM) **(G)**, and then exposed to the control (300 mOsM) condition or the 485 mOsM HGH solution for 24 h. Statistical differences were assessed by two-way ANOVA followed by Tukey's *post-hoc* test. ^**^P < 0.01 against control condition (300 mOsM). Graph bars show the mean ± *SD* (*N* = 5 independent cultures).

NOX activity is triggered by several phosphorylations in serine residues, as mediated by PKC (el Benna et al., 1994; Park and Babior, 1997; Dang et al., 1999; Lopes et al., 1999; Babior, 2002, 2004; Simon and Stutzin, 2008). Therefore, tests were performed to determine if PKC activity participated in HGH-induced HPMC death. For this, HPMC were pre-incubated with a non-selective PKC inhibitor, BIM, and cells were then exposed to the HGH solution. BIM treatment decreased the HPMC death generated by HGH exposure (Figure 5A). Conventional PKC (cPKC), composed of PKCα, PKCβ, and PKCγ, is a PKC isoform that phosphorylates NOX (el Benna et al., 1994; Park and Babior, 1997; Lopes et al., 1999; Babior, 2002, 2004; Simon and Stutzin, 2008). Considering this, Gö 6976 was used to selectively inhibit cPKC, thus allowing for determinations of if this PKC isoform is involved in HGH-induced HPMC death. In accordance with the results observed using BIM, Gö 6976-treated cells were resistant to the HPMC death induced by HGH exposure (Figure 5B). cPKC are activated by DAG and Ca^2+^, and HGH exposure increased intracellular Ca^2+^ content (Figures 2A,B). On the other hand, DAG is generated by the actions of PLC. Therefore, the PLC inhibitor U73122 was used to assess the involvement of PLC activity. U73122 inhibited HGH-induced HPMC death (Figure 5C). Generally, PLC is activated by G-protein actions. HPMC incubated with a non-selective G-protein inhibitor were resistant to death as induced by the HGH solution (Figure 5D). These results suggest that the G-protein/PLC/cPKC pathway participates in the HPMC death induced by the HGH condition.

Previous studies have demonstrated that hyperglycemia induces PI3-K/Akt pathway activation to mediate several cellular processes (Hao et al., 2011; Zhao et al., 2012). Additionally, the PI3-K/Akt pathway promotes cell death in numerous cell types, often through mechanisms mediated by ROS overproduction (Zhang and Yang, 2013). Considering this information, assessments were carried out to determine if the PI3-K/Akt pathway participates in HGH-induced HPMC death. For this, HPMC were pre-incubated with a non-selective PI3-K inhibitor, Ly294002, and cells were then subjected to the HGH condition. The Ly294002-treated mesothelial cells showed decreased HGH-induced HPMC death (Figure 5E). Additionally, mesothelial cells were incubated with a selective inhibitor of Akt, API-2, and the effect of HGH exposure was evaluated. The results showed that Akt inhibition provoked a partial, but significant, inhibition of HPMC death induced by the HGH condition (Figure 5F). These results are in agreement with previously showed in apoptotic rat PMC (Kaifu et al., 2009). PI3-K/Akt signaling is triggered by the activation of the tyrosine-kinase receptor, and HPMC cultured with a non-selective inhibitor of the tyrosine-kinase receptor were resistant to cell death induced by the HGH solution (Figure 5G). These findings suggest that the tyrosine-kinase receptor/PI3-K/Akt pathway is involved in HGH-induced HPMC death.

## Discussion

High-glucose hypertonic solutions are used in chronic PD as a replacement therapy during terminal renal failure. Considering that this clinical strategy is as effective as hemodialysis, in addition to providing more comfort and mobility to patients, PD is the treatment of choice for several types of renal patients (Teixeira et al., 2015). Unfortunately, PD that uses HGH solutions has, in several patient cases, resulted in severe peritoneal damage associated with mesothelial cell death (Teixeira et al., 2015). However, the specific underlying molecular mechanism involved in HGH solution-induced HPMC death is unknown.

The results of this study demonstrate that HPMC exposed to the HGH solution exhibit extensive cell death. HGH solution challenges induced increased intracellular Ca^2+^ and Na^+^ levels via ion influxes possibly mediated by ion channels. Interestingly, HGH-induced HPMC death was dependent on intracellular Ca^2+^ and Na^+^ increases and was inhibited by both Ca^2+^ and Na^+^ channels blockers. Furthermore, HGH exposure generated increased intracellular ROS levels. In fact, HPMC death was induced by oxidative stress in a dose-dependent manner. In accordance with these results, HGH-induced HPMC death was inhibited by antioxidant or reducing agents, suggesting that the HPMC death as induced by the HGH solution is dependent on the generation of oxidative stress. Related to this, NOX2 was identified as a probable main intracellular source for the ROS that mediated HGH-induced HPMC death. Additionally, HGH-induced HPMC death was also dependent on NO generated by NOS activity. Besides this, the obtained results demonstrated that the G-protein/PLC/cPKC and tyrosine-kinase receptor/PI3K/Akt intracellular signaling pathways were crucial for mediating the HPMC death induced by the HGH solution.

In particular, the 485 mOsM HGH solution, which is comparable to a commercial solution of 4.25% dextrose, induced significant HPMC death. In contrast, the 345 and 395 mOsM HGH solutions did result in any deleterious effects. These data indicate that only the highest HGH solution generates HPMC death. However, considering that the presently performed experiments only considered a 24 h period, it is plausible to postulate that over a prolonged time-course treatment, the 345 and 395 mOsM HGH solutions might also generate HPMC death. Further experiments must be performed to test if this is the case. Interestingly, experiments that used sorbitol instead of glucose, which were performed to maintain hypertonicity in the absence of glucose participation, revealed that sorbitol-treated HPMC did not exhibit cell death. These results indicate that a high glucose concentration, but not hypertonicity, is a crucial and obligatory factor for causing HPMC death. Related to this, new PD solutions are under assessment that do not contain glucose-osmotic agents, instead using icodextrin or amino acids, a neutral pH, and low levels of glucose degradation products (GDP) to prevent detrimental effects to HPMC (Ha et al., 2000; Chan et al., 2003; Mortier et al., 2004; García-López et al., 2012). Additionally, solutions with high glucose concentrations generate GDP. Often, GDP are generated as a consequence of heat sterilizing peritoneal dialysis (PD) fluids. GDP triggers the formation of advanced glycation end-products in the peritoneal cavity, as well as producing extensive cytotoxicity and cell transformation by means of a poorly understood mechanism (Witowski and Jörres, 2000; Schwenger, 2005; Oh et al., 2010; Pischetsrieder and Gensberger, 2012).

The expression and activities of the Na^+^-glucose cotransporters 1 and 2 (SGLT1 and SGLT2) regulate glucose transport (Vallon, 2010; Hummel et al., 2011). At the same time, glucose transport is involved in several important pathologies (Koepsell, 2017). Current evidence shows that SGLT1, GLUT1, and GLUT3 are expressed in differentiated mesothelial cells (Schröppel et al., 1998) and are directly involved in high glucose dialysate-induced peritoneal fibrosis (Hong et al., 2016). Of note, intracellular hypertonicity is responsible for water movement in association with Na^+^-glucose cotransport (Charron et al., 2006). Considering this, it is possible that the Na^+^ influx mediated by SGLT due to the increased glucose gradient generates HPMC membrane depolarization with the subsequent activation of sodium and calcium channels. Alternatively, the glucose influx through SGLT or GLUT proteins could shift the ATP/ADP equilibrium promoting the activation of nucleotide-dependent sodium and calcium channels (Gafar et al., 2016). Further experiments must be performed using SGLT and/or GLUT inhibitors to evaluate these considerations.

Epithelial-to-mesenchymal transition (EMT) occurs in a significant portion of samples from patients treated with PD using HGH solutions (Kalluri and Neilson, 2003; Yáñez-Mó et al., 2003; Kalluri and Zeisberg, 2006; Aroeira et al., 2007; Del Peso et al., 2008; Goffin, 2008), and this conversion could contribute to a total failure of the peritoneal capacity for ultrafiltration. Consequently, the EMT process is the most studied feature involved in peritoneal loss-of-function (Aroeira et al., 2007; Del Peso et al., 2008; Goffin, 2008). Mesothelial cells exposed to TGF-ß1 undergo EMT-mediated fibrosis, thereby becoming a possible source of myofibroblasts in peritoneal fibrosis (Szeto et al., 2006; Aroeira et al., 2007; Lv et al., 2011; García-López et al., 2012; Krediet and Struijk, 2013; Kokoroishi et al., 2015). Hyperglycemic conditions can generate increased expressions of TGF-β1 and pro-inflammatory cytokines in peritoneal mesothelial cells (Shanmugam et al., 2003; Luo et al., 2012; Kumar et al., 2014; Kang et al., 2015; Kokoroishi et al., 2015; Li et al., 2016). Interestingly, a number of cytokines are induced by high glucose levels (Shanmugam et al., 2003; Kumar et al., 2014). TGF-β1, ROS, and pro-inflammatory cytokines are potent inducers of the extracellular matrix protein overexpression that overwhelms degradation capacities, and the peritoneal damage induced by PD is associated with a submesothelial increase of extracellular matrix proteins (Davies et al., 2001; Williams et al., 2002; Mortier et al., 2004; Goffin, 2008). Supporting this, PD patient biopsies have reported that peritoneal membrane thickness is increased by 54% (Yáñez-Mó et al., 2003; Aroeira et al., 2007; Del Peso et al., 2008; Goffin, 2008).

**Figure 6:**
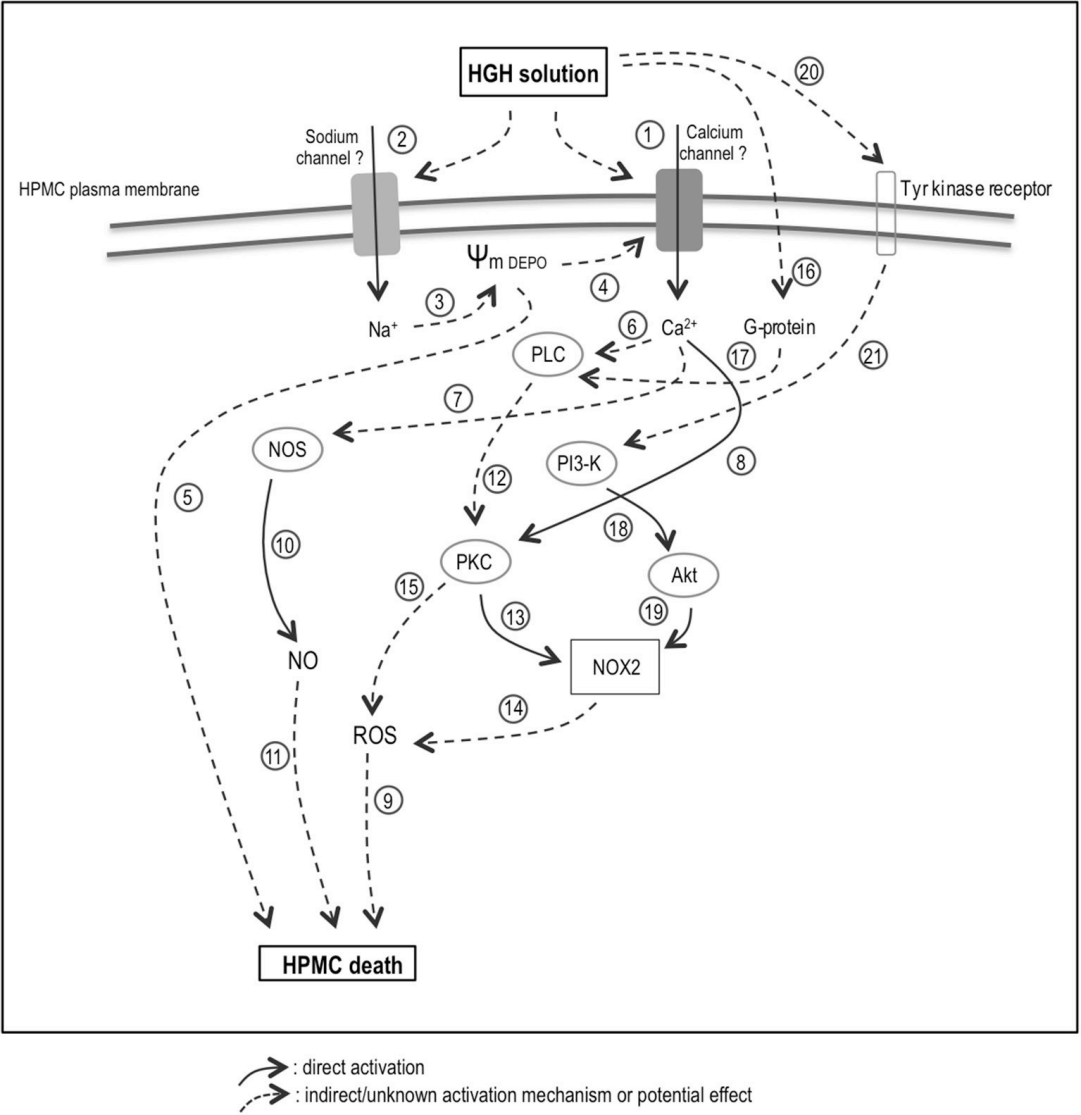
Proposed model for the HGH solution-induced HPMC death. HGH solution increases of Ca^2+^ (1) and Na^+^ (2) levels are potentially dependent on ion channel activities. A sodium influx could generate membrane depolarization (3), which can induce voltage-activated calcium channel opening (4) and cell death (5). Intracellular calcium may activate enzymes, including PKC (6), PLC (7), and NOS (8). Increased ROS level could trigger HGH-induced HPMC death (9), and NOS-dependent NO generation would be relevant in inducing HPMC death (9–10). HGH-induced HPMC death could be carried out through the PLC/PKC/NOX/ROS signaling pathway (12–15). In line with this, NOX2 might be involved in both HGH-induced ROS production (14) and HPMC death (9). Furthermore, PKC activation may occur as a consequence of PLC-mediated G-protein activation (16–17). The PI3K/Akt pathway could also involved in the HPMC death induced by HGH exposure (18–19), and would be mainly activated through the activation of tyrosine-kinase receptors (20–21). HGH, high-glucose hypertonic; NOS, nitric oxide synthase; NO, nitric oxide; H_2_O_2_, hydrogen peroxide; HPMC, human peritoneal mesothelial cells; PKC, protein kinase C; NOX, nicotinamide adenine dinucleotide phosphate-oxidase; PI3-K, phosphatidylinositol 3-kinase; Akt, protein kinase B. Solid arrow: direct activation. Discontinuous arrow: indirect/unknown activation mechanism or potential effect.

The elevated intracellular Ca^2+^ and Na^+^ levels, as induced by the HGH solution, are possibly dependent on ion channel activities (Figure 6, No. 1 and 2). In fact, an imbalance in intracellular ions is a hallmark of cell death (Henriquez et al., 2008; González-Mateo et al., 2009; Becerra et al., 2011; Nuñez-Villena et al., 2011). It is generally accepted that a sodium influx generates cell death-mediated membrane depolarization (Figure 6, No. 3). This membrane depolarization can induce voltage-activated calcium channel opening, resulting in an influx of Ca^2+^ (Figure 6, No. 4). Use of calcium channel blockers in the conducted analyses suggests the participation of TRP channels, P2X-type purinergic channels, and connexin. However, further experiments are needed to establish the participation of one or several types of channels. Additionally, severe membrane depolarization can induce cell death by enhancing swelling (Figure 6, No. 5). On the other hand, intracellular calcium may activate several cytoplasmic enzymes, including PKC, PLC, and NOS (Figure 6, No. 6–8; Lecca et al., 2012; Figueroa et al., 2013; Echeverría et al., 2014a,b; Retamal et al., 2015; García et al., 2016).

Hyperglycemia damages many tissues through several mechanisms, with elevated glucose concentrations increasing the ROS levels in peritoneal HPMC (Taylor et al., 1992; Ha and Lee, 2000; Tarng, 2002). Indeed, ROS production by NOX2 can be stimulated by hyperglycemia in cardiomyocytes through SGLT1 (Balteau et al., 2011) and ROS may amplify intracellular signaling when glucose is elevated (Taylor et al., 1992; Ha and Lee, 2000; Tarng, 2002; Zuo et al., 2011). Furthermore, ROS generation frequently involves the production of ${\text{O}}_{2}^{•-}$, which is rapidly converted into H_2_O_2_ through the actions of superoxide dismutase. Subsequently, H_2_O_2_ could be either reduced to a hydroxyl radical, generating a consequent increase in intracellular oxidative stress, or converted into H_2_O by catalase, thereby decreasing oxidative stress (Dröge, 2002). Accordingly, EMT, as well as endothelial-mesenchymal transition, are both stimulated by ROS (Montorfano et al., 2014; Mahalingaiah et al., 2015; Pérez et al., 2016). Increased oxidative stress creates a suitable environment for triggering mesenchymal conversion into epithelial and endothelial cells, and the induction of EMT in patients treated with PD is associated with high glucose concentrations through a mechanism mediated by ROS generation (Davies et al., 2001; Lee et al., 2004; Rhyu et al., 2005; Książek et al., 2007a,b). Therefore, elevated intracellular ROS levels appear crucial for eliciting HGH-induced HPMC death (Figure 6, No. 9). This finding is in accordance with previously reported results in several cell types, including in endothelial, neuronal, and epithelial cells (Kumaran and Shivakumar, 2002; Hecquet and Malik, 2009; Coombes et al., 2011). Additionally, NOS activity appeared crucial for eliciting HGH-induced HPMC death, suggesting that NOS-dependent NO generation is relevant in inducing HPMC death (Figure 6, No. 9 and 10). This finding is reasonable as NO can react with ROS to generate the highly reactive radical NO^•−^ (Dröge, 2002).

The presently obtained results indicate that HGH-induced HPMC death is potentially carried out through the signaling pathway PLC/PKC/NOX/ROS (Figure 6, No. 12–14). On the other hand, alternative pathways could elicit ROS generation upon PKC activation (Figure 6, No. 15). Previous studies report that high glucose levels induce ROS generation through the activation of PKC, NAD(P)H oxidase, and mitochondrial metabolism-promoting fibronectin expression (Ha and Lee, 2000; Lee et al., 2004). Although the present results found cPKC to be the kinase that activates NOX-generated ROS, additional PKC isoforms, such as novel and atypical subfamilies, could also activate NAD(P)H oxidase. Using siRNA-based technology, the NOX2 isoform was found involved in both HGH-induced ROS production and HPMC death (Figure 6, No. 9 and 14). Similarly, in endothelial cells and macrophages, NOX2 is the preponderant subunit in producing oxidative stress (Babior, 2002; Simon and Fernández, 2009). The current results additionally suggest that PKC activation may occur as a consequence of PLC-mediated G-protein activation (Figure 6, No. 16 and 17). However, the specific identity of the activated G-protein remains to be clarified.

The PI3K/Akt pathway is also involved in the HPMC death induced by HGH exposure (Figure 6, No. 18 and 19). Akt can phosphorylate NOX in the absence of PKC, thereby inducing activation (Chen et al., 2003; Hoyal et al., 2003; Figure 6, No. 19). Human lung epithelial cells exposed to high glucose exhibit cell invasion and metastasis via the participation of ROS and the TGF-β1/PI3K/Akt signaling pathway (Kang et al., 2015). In turn, the PI3K/Akt pathway involved in the HGH solution-induced HPMC death was mainly activated through the activation of tyrosine-kinase receptors (Figure 6, No. 20 and 21). However, the precise mechanism involved in this process is not yet well-understood.

The currently reported results represent a relevant advancement toward fully understanding the initial steps of the mechanism underlying mesothelial cell failure. This information should be considered in the development of a more efficient strategy for treating PD patients undergoing renal failure.

## Author contributions

FS, PT, CE, RA, SG, AV, AP, MS, MA, ES, and RT, all of them revised and edited critically this manuscript. FS, PT, RA, CE, AP, MS, MA, ES, and RT, participated in research design. FS, PT, RA, CE and RT, conducted experiments and performed data analyses. FS, PT, and RT contributed to the figure design. FS, PT, SG, AV, and RT wrote the paper.

### Conflict of interest statement

The authors declare that the research was conducted in the absence of any commercial or financial relationships that could be construed as a potential conflict of interest.
